# MicroRNA-155-5p is a key regulator of allergic inflammation, modulating the epithelial barrier by targeting PKIα

**DOI:** 10.1038/s41419-019-2124-x

**Published:** 2019-11-25

**Authors:** Xiaoyu Wang, Yanyan Chen, Weiyuan Yuan, Lu Yao, Siqi Wang, Zhirong Jia, Peng Wu, Lianqu Li, Pan Wei, Xiaotong Wang, Min Hong

**Affiliations:** 1 Jiangsu Key Laboratory for Pharmacology and Safety Evaluation of Chinese Materia Medica, School of Pharmacy, Nanjing University of Chinese Medicine, Nanjing, 210023 China; 2 Present Address: School of Pharmacy, Shandong University of Traditional Chinese Medicine, Jinan, 250355 China

**Keywords:** Inflammation, Atopic dermatitis

## Abstract

Recent studies have demonstrated that microRNA-155-5p (miR-155-5p) plays an essential role in the regulation of allergen-induced inflammation and is overexpressed in the skin of patients with atopic dermatitis (AD), although the mechanism is unknown. In this study, silencing miR-155-5p attenuated the thickening of the epidermis in AD model and reduced the infiltration of inflammatory cells and the secretion of Th2 cytokines. Protein kinase inhibitor α (PKIα) was identified as a direct target of miR-155-5p and correlated negatively with miR-155-5p in our AD model. Fluorescence in situ hybridization showed that miR-155-5p-expressing cells were predominantly present in the epidermis. When epithelial cells were transfected with an miR-155-5p inhibitor, the expression of PKIα, occludin, and CLDN16 increased and that of TSLP decreased significantly, whereas the overexpression of miR-155-5p resulted in the opposite changes. The increased expression of PKIα and tight junction (TJ) proteins, with reduced TSLP and IL-33, was also detected in miR-155-5p-blocked mice, in both the initial and elicitation stages of AD. The expression of TJ proteins also decreased when cells were transfected with PKIα siRNA. TJ proteins increased and TSLP and IL-33 decreased significantly after the overexpression of PKIα. Our data provide the first evidence that miR-155-5p is critical for the allergic inflammation in a mouse model of AD by directly regulating PKIα and thus epithelial TJ expression. These findings suggest new therapeutic strategies that target miR-155-5p in patients with allergic disorders.

## Introduction

Atopic dermatitis (AD) is a common chronic inflammatory disease characterized by intense, dry pruritus, inflamed skin, and a relapsing and often chronic course^1,2^. Many of these features are attributed to an impaired skin barrier and a dysregulated immune response to a variety of triggers^3^.

The skin is the only epithelial surface that has two barrier structures. The stratum corneum and tight junctions (TJs) form a physical barrier that protects the body from inhaled harmful substances. Disturbance of the epithelial barrier is now recognized as a common feature in many inflammatory diseases, including asthma^4,5^, food allergies, inflammatory bowel disease^6^, sinusitis, and AD^7,8^. A defect in the barrier increases the penetration of allergens, microbes, and irritants into the dermis. We have previously reported the restoration of the epithelial barrier function by inhibiting the expression of key promoters of allergy (thymic stromal lymphopoietin [TSLP] and interleukin 33 [IL-33]), which contribute to the Th2 immune response observed in early AD^9^. Findings support the notion that a deficiency in the primary skin barrier is central to the initiation and progression of AD^10^.

MicroRNAs (miRNAs) belong to a growing family of small noncoding RNAs that alter gene expression by inhibiting mRNA translation or initiating the degradation of mRNA targets, thereby regulating cellular responses^11,12^. miR-155-5p was among the first miRNAs linked to inflammation because it potently upregulates multiple immune cells, including lymphocytes, dendritic cells, and macrophages^13–15^. Recent studies have demonstrated that miR-155-5p plays an essential role in the regulation of allergen-induced inflammation^16^, and several have implicated it in the pathogenesis of chronic skin and airway inflammation^17–19^. miR-155-5p is upregulated in an asthma model^20^, and is a critical regulator of type 2 innate lymphoid cells and IL-33 signaling in allergic airway inflammation^21^. Transcriptomics has also identified a critical role for Th2-cell-intrinsic miR-155-5p in mediating allergic inflammation^22^. miR-155-5p is also overexpressed in the skin of patients with AD^23^ and the nasal mucosa of patients with allergic rhinitis or asthma^24^.

MiRNAs can regulate the expression of TJ proteins and thus modulate their epithelial barrier function. Claudin-1 (CLDN-1) expression is downregulated by an increase of miR-155-5p in ovarian cancer cells^25^. In intestinal epithelial cells, miR-155-5p is induced by tumor necrosis factor α (TNF-α), which leads to the downregulated expression of zonula occludens 1 (ZO-1) and E-cadherin. These data emphasize the key role of miR-155-5p in the regulation of the barrier function^26^. However, there are only limited data on the functional role of miR-155-5p in the skin of AD patients.

We previously identified miR-155-5p as upregulated in a mouse model of recurrent AD with an miRNA microarray analysis, and showed that protein kinase A inhibitor α (PKIα) is a potential target of miR-155-5p^27^. PKIα is a member of the protein kinase A inhibitor (PKI) family and is expressed in the human heart, brain, skin, and 11 other tissues^28^. Previous studies have shown that PKIα is altered in AD patients^29^, and that protein kinase inhibitors prevent the disruption of epithelial TJ proteins^30^. However, the importance of miR-155-5p in the pathogenesis of AD remains unknown. In this study, we investigated the role of miR-155-5p in AD, and identified the specific targets of miR-155-5p to examine the possible regulatory effects of miR-155-5p on TJs, thus clarifying the pathogenesis of AD.

## Results

### miR-155-5p predominantly increased in the epithelium in AD model and HaCaT cells

**Fig. 1:**
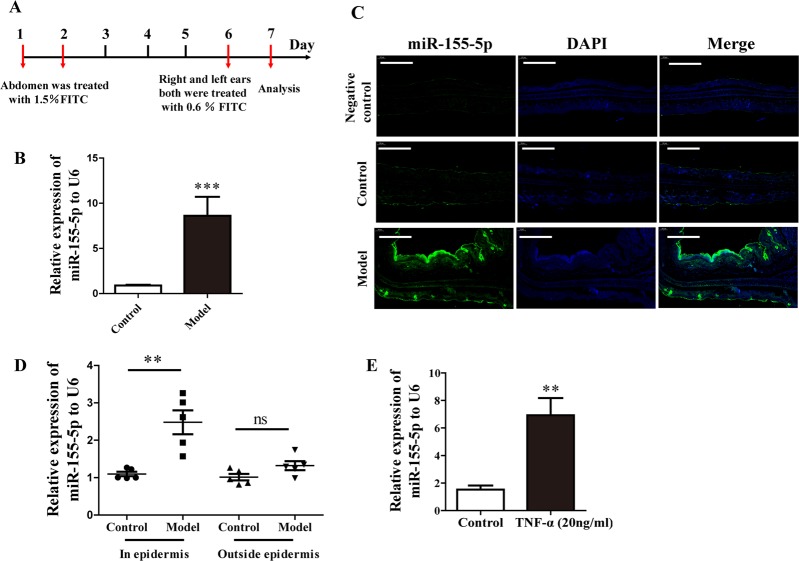
miR-155-5p was expressed predominantly in the epithelium in the AD model and HaCaT cells. **a** Overview of the protocol used to establish the FITC-induced AD mouse model. **b**, **c** miR-155-5p expression in mouse ears was analyzed with PCR and fluorescence in situ hybridization (green: miR-155-5p; blue: DAPI-labeled nuclei; scale bar: 200 μm). **d** miR-155-5p expression in the epidermis and outside the epidermis were detected by PCR. **e** miR-155-5p expression in HaCaT cells was measured after TNF-α stimulation for 24 h (mean ± SD, *n* = 6; *P < 0.05, **P *<* 0.01 versus control, ns: not significantly).

A mouse model of AD was established to investigate the expression of miR-155-5p in vivo (Fig. 1a). qPCR showed that the expression of miR-155-5p was significantly increased in these mice (Fig. 1b). Changes in miR-155-5p expression were detected with fluorescence in situ hybridization. A scrambled miR was used as the negative control. The scrambled control probe showed no significant staining in the control group or the model group. miR-155-5p-expressing cells were predominantly found in the epidermis, and miR-155-5p expression was clearly increased in the model group (Fig. 1c). The expression of miR-155-5p in the epidermis was also detected with PCR. miR-155-5p expression was significantly higher in the epidermis of the AD model group than in that of the control group. For comparison, we also detected the expression of miR-155-5p in other tissues outside the epidermis, but the changes in miR-155-5p were not significant (Fig. 1d). When HaCaT cells were stimulated with TNF-α, their expression of miR-155-5p increased significantly (Fig. 3e).

### miR-155-5p is required for allergic inflammation in mice AD

**Fig. 2:**
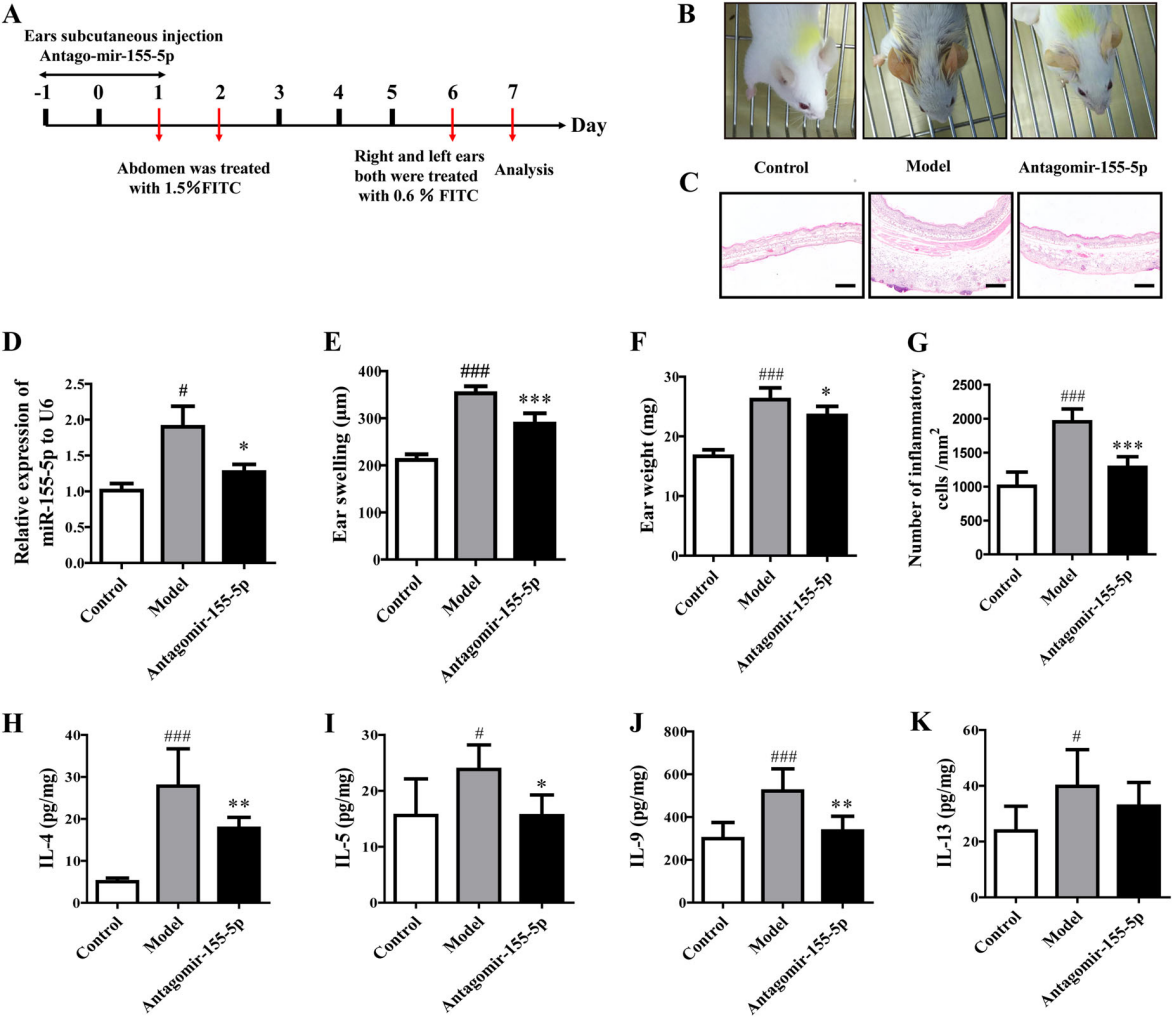
Silencing miR-155-5p inhibited allergic inflammation in the AD model. **a** Flow chart of antagomir-155-5p administration to AD murine model. **b** Representative images of mouse ears on day 6 after challenge with FITC. **c**, **g** Histopathological changes examined with H&E staining (*n* = 5; magnification: ×100; scale bar: 200 μm). The number of inflammatory cells was quantified with the Mantra Quantitative Pathology Workstation. **d** Expression of miR-155-5p was detected with PCR. **e**, **f** Ear thicknesses and ear weights were measured on day 7. **h**–**k** Th2 cytokine production was measured in ear homogenates with ELISAs (mean ± SD; *n* = 7; ^#^*P* < 0.05, ^###^*P* < 0.001 versus control, *P < 0.05, **P < 0.01, ***P < 0.001 versus model).

To investigate whether miR-155-5p is required for the development of inflammation in AD, mice were treated for three consecutive days with an injection of an miR-155-5p inhibitor or a control inhibitor (antagomir-155-5p group) into the ear on days −1 to 1, based on the AD model (Fig. 2a). Allergic inflammation was confirmed on day 7. Red and swollen ears were observed in the model mice but not in the control mice, and the pathology was clearly abrogated in the antagomir-155-5p-treated group (Fig. 2b). Treatment with antagomir-155-5p before fluorescein isothiocyanate (FITC) sensitization effectively attenuated the thickening of the epidermis and the infiltration of inflammatory cells in the AD mice (Fig. 2c, g). miR-155-5p expression was significantly reduced when the mice were treated with the miR-155-5p inhibitor before FITC sensitization (Fig. 2d). We confirmed the marked alleviation of inflammation by antagomir-155 in mice with FITC-induced AD by measuring the ear swelling and ear weights (Fig. 2e, f). The levels of Th2 cytokines (IL-4, IL-5, IL-9, and IL-13) were significantly elevated in the AD model, but were reduced in the antagomir-155-5p-treated mice (Fig. 2h–k). These observations suggested that miR-155-5p was required to establish the AD-associated inflammation induced by allergen challenge.

### PKIα is a specific target of miR-155-5p

**Fig. 3:**
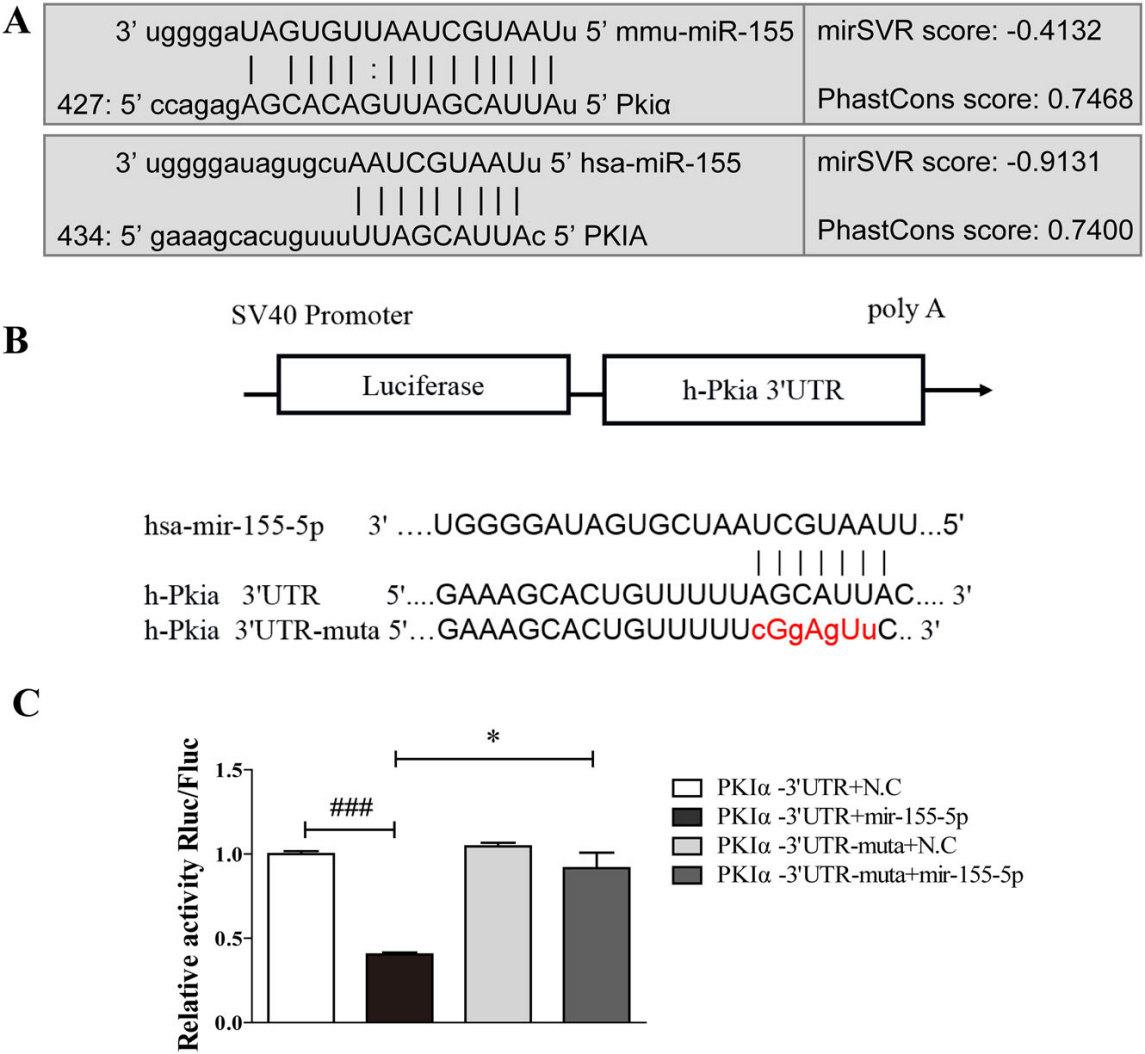
PKIα mRNA is a specific target of miR-155-5p. **a**, **b** Region of the human PKIα mRNA 3′UTR predicted to be targeted by miR-155-5p (miRanda) and the mutation introduced into the seed region of the miR-155-5p target site (PKIα-mut). **c** 293T cells were cotransfected with luciferase reporter vectors (PKIα-3′UTR or mut-PKIα-3′UTR, and miR-155-5p mimic or miR-155-5p control). Luciferase values were normalized to the activity of the *Renilla* control luciferase. The luciferase activity ratio of each construct was calculated with a luminometer (mean ± SD; *n* = 6; ^###^*P* < 0.001 versus PKIα-3′UTR + NC; *P < 0.05 versus PKIα-3′UTR + miR-155 mimic).

The miRanda and TargetScan databases were used to predict potential targets of miR-155-5p^27^. Based on the highest scores in the databases, we selected PKIα as a potential target. The putative miRNA target site was identified in the PKIα mRNA 3′ untranslated regions (3′UTRs) of the human and mouse transcripts (Fig. 3a). To confirm that PKIα is an actual target of miR-155-5p, 293T cells were cotransfected with a luciferase reporter containing the wild-type or mutant PKIα 3′UTR miR-155-binding site and the miR-155-5p expression vector or the negative control (NC) vector. The activity of the luciferase reporter containing the wild-type PKIα 3′UTR was significantly inhibited when cotransfected with miR-155-5p, whereas the activity of the reporter containing the mutated PKIα 3′UTR site was unaffected by cotransfection with miR-155-5p (Fig. 3b, c). These results demonstrated that miR-155-5p targeted PKIα mRNA by specifically binding to its 3′UTR.

### PKIα downregulation in the AD model and HaCaT cells

**Fig. 4:**
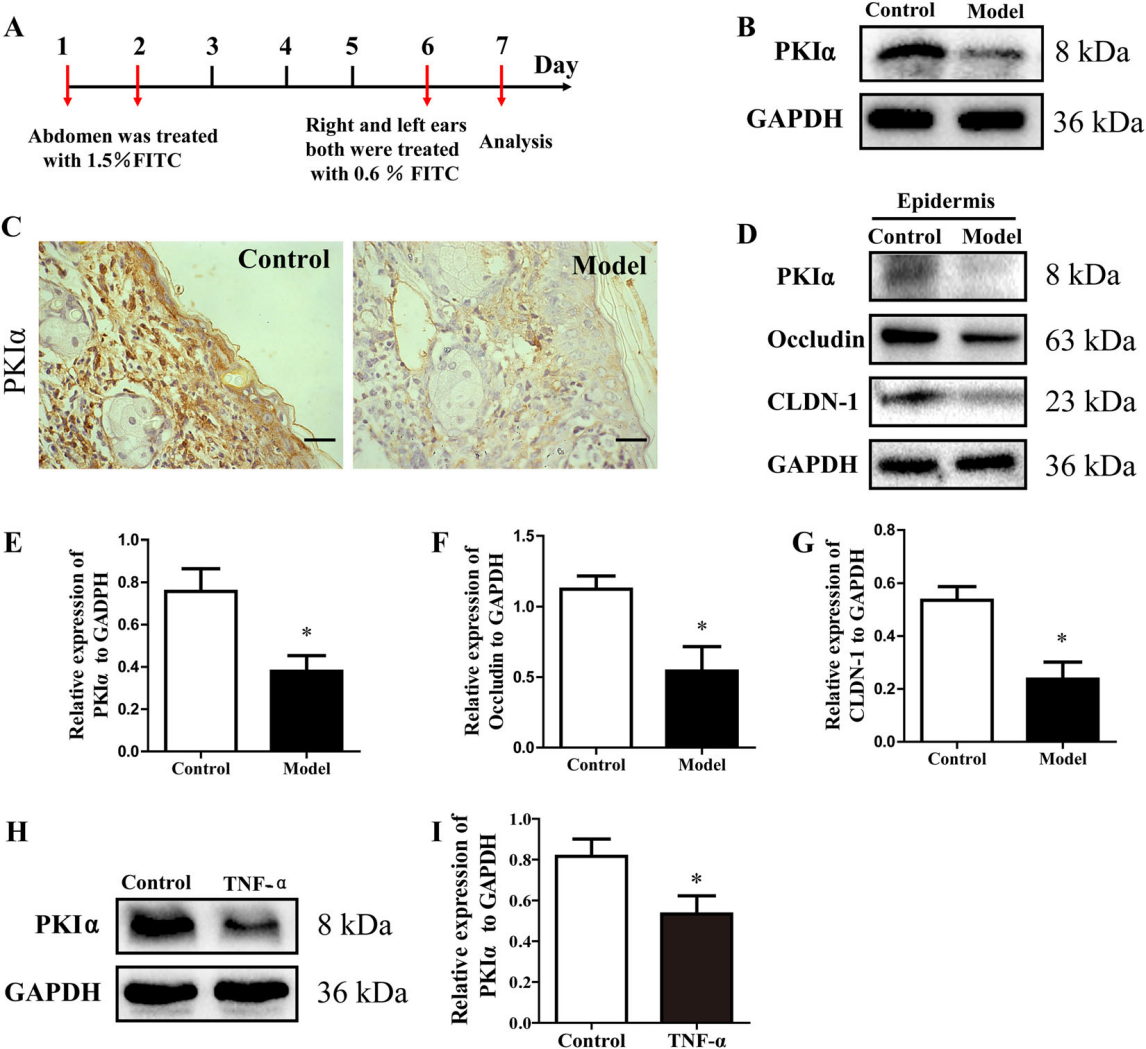
PKIα downregulation in the AD model and HaCaT cells. **a**–**c** PKIα expression was analyzed with immunohistochemistry and western blotting (*n* *=* 5; scale bar: 50 μm). **d**–**g** PKIα, CLDN-1, and Occludin expression levels in the epidermis of ears were detected by western blotting. The expression levels relative to GAPDH were quantified with a ChemiScope analysis (mean ± SD; *n* = 3; *P < 0.05 versus control). **h**, **i** PKIα expression in cells was analyzed with western blotting. Its expression relative to GAPDH was quantified with a ChemiScope analysis (mean ± SD, *n* = 6; *P < 0.05, **P *<* 0.01 versus control, ns: not significantly).

PKIα expression was greatly reduced in the AD model compared with that in the control group (Fig. 4a–c). The expression of PKIα, occludin, and CLDN-1 in the epidermis was detected with western blotting. All three proteins were significantly reduced in the AD model group compared with the control group (Fig. 4d–g). Similarly, when HaCaT cells were stimulated with TNF-α, PKIα decreased significantly (Fig. 4h, i).

### Inhibited or overexpressed miR-155-5p altered the expression of PKIα, TJ proteins, TSLP, and IL-33 in HaCaT cells in vitro

**Fig. 5:**
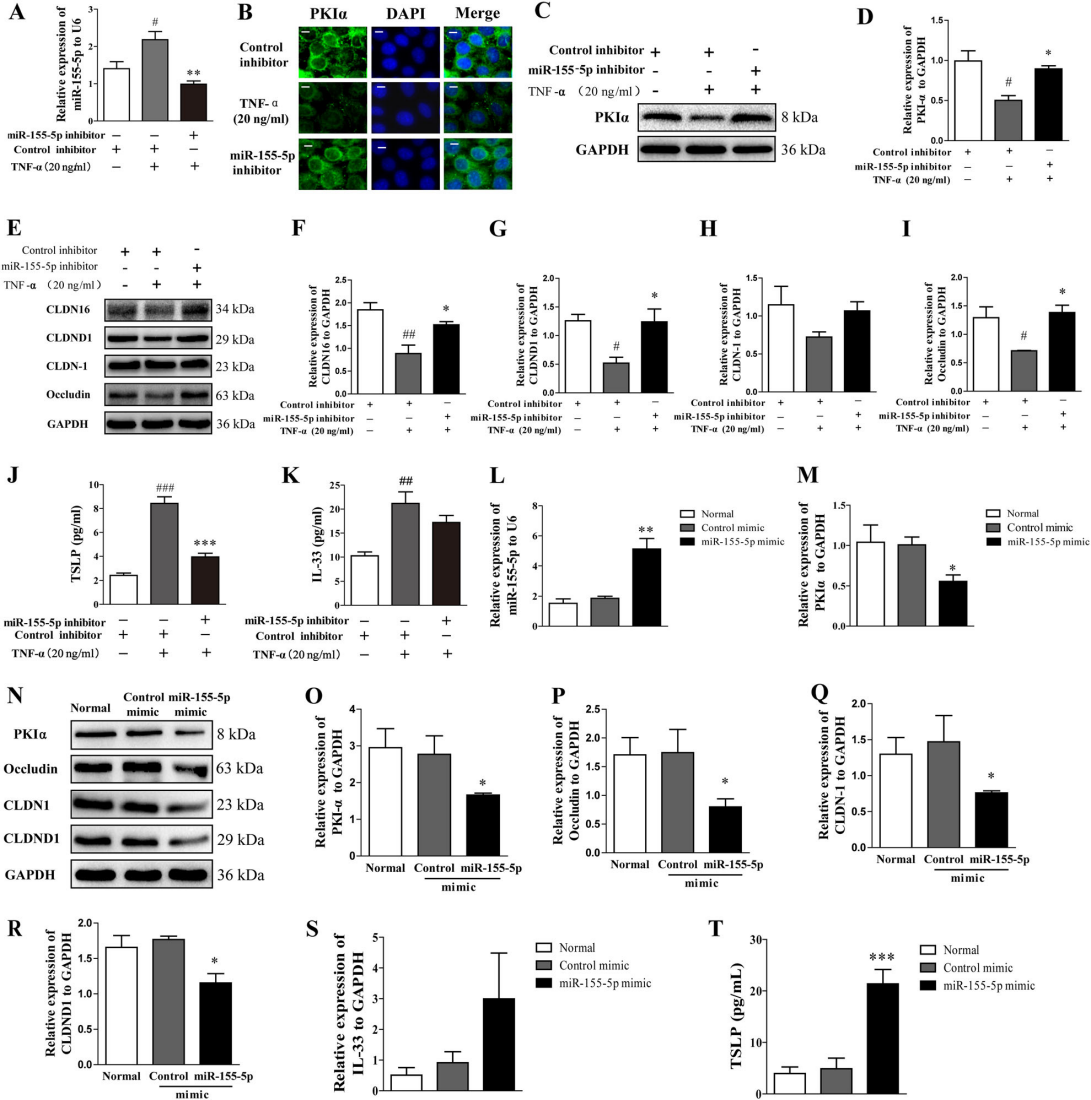
Inhibited or overexpressed miR-155-5p alters PKIα, TJ proteins, TSLP, and IL-33 expression in HaCaT cells. **a** The effect of transfecting the cells with an miR-155-5p inhibitor was analyzed with PCR. **b–d** PKIα expression was analyzed with immunofluorescence (*n* *=* 4; magnification: ×200; scale bar: 100 μm) and western blotting. The expression levels relative to GAPDH were quantified with a ChemiScope analysis (*n* = 3). **e–i** TJ protein expression was analyzed with western blotting. The expression levels relative to GAPDH were quantified with a ChemiScope analysis (*n* = 3). **j, k** TSLP and IL-33 production was measured with ELISAs (mean ± SD; *n* = 6; ^#^*P* < 0.05, ^##^*P* < 0.01, ^###^*P* < 0.001 versus control, **P < 0.01, ***P < 0.001 versus TNF-α). **l** Effect of transfecting the cells with an miR-155-5p mimic was analyzed with PCR. **m** PKIα expression was analyzed with PCR. **n–r** PKIα, occludin, CLDN1, and CLDND1 expression was analyzed with western blotting. The expression levels relative to GAPDH were quantified with a ChemiScope analysis (*n* = 3). **s, t** TSLP and IL-33 expression was analyzed with ELISA and PCR, respectively (mean ± SD; *P < 0.05, **P < 0.01, ***P < 0.001 versus mimic control). The data are representative of three independent experiments.

To investigate the effects of miR-155-5p on PKIα, HaCaT cells were transfected with a miR-155-5p inhibitor. TNF-α significantly increased the level of miR-155-5p, which was effectively downregulated by the miR-155-5p inhibitor (Fig. 5a). The expression of PKIα and the TJ proteins (CLDN16, CLDND1, and occludin) was clearly reduced after treatment with TNF-α, but restored by the miR-155-5p inhibitor (Fig. 5b–i). The cells treated with the miR-155-5p inhibitor showed significantly reduced TSLP expression and a trend toward lower expression of IL-33 (Fig. 5j, k).

The effects of miR-155-5p overexpression on PKIα, TJ proteins, and IL-33 were investigated by transfecting HaCaT cells with an miR-155-5p mimic (Fig. 5l). The expression of PKIα and TJ proteins (CLDN-1, CLDND1, and occludin) was clearly reduced by the overexpression of miR-155-5p (Fig. 4m–r). TSLP was significantly increased in the miR-155-5p mimic group (Fig. 5t).

### Silencing miR-155-5p restored PKIα TJ protein expression and reduced TSLP and IL-33 production in different stages of AD model

**Fig. 6:**
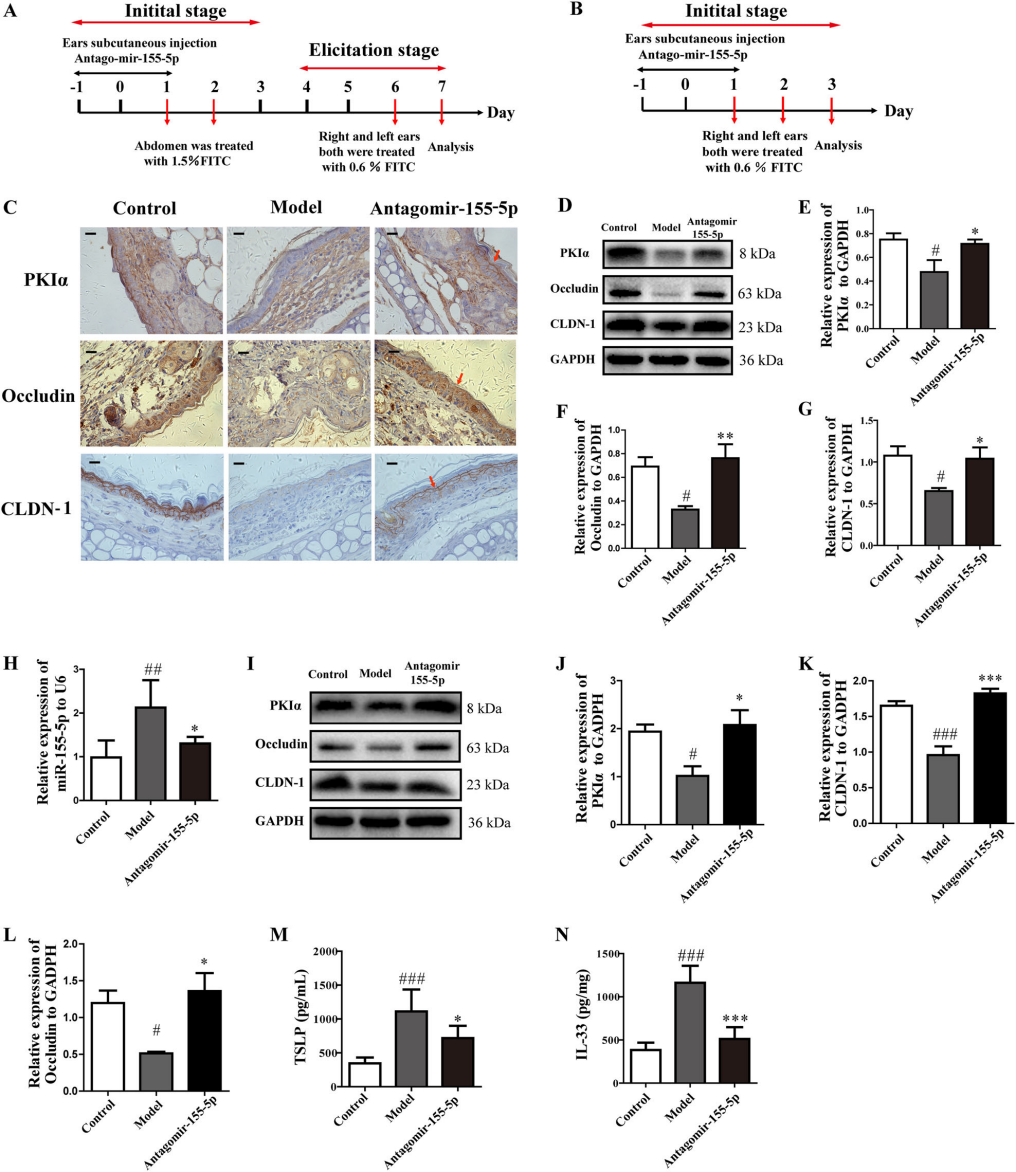
Silencing miR-155-5p restored PKIα and TJ protein expression and reduced TSLP and IL-33 production in different stages of the AD model. **a**, **b** Overview of the protocol used to establish the elicitation and initial stages of the FITC-induced AD mouse model. **c**, **d** PKIα, CLDN-1, and occludin expression was analyzed with immunohistochemistry (*n* = 5; magnification: ×630; scale bar: 50 μm) and with western blotting in the elicitation stage of the AD model. **e**–**g** PKIα, CLDN1, and occludin expression was quantified relative to GAPDH expression with a ChemiScope analysis. **h**, **i** Expression of miR-155-5p was detected with PCR and that of PKIα, CLDN-1, and occludin was analyzed with western blotting in the initial stage of the AD model. **j**–**l** PKIα, CLDN-1, and occludin expression was quantified relative to GAPDH expression with a ChemiScope analysis (*n* = 4). **m**, **n** TSLP and IL-33 production in ear homogenates in the initial stage of the AD model was measured with ELISAs (mean ± SD; *n* = 6; ^#^*P* < 0.05, ^##^*P* < 0.01, ^###^*P* < 0.01 versus control, *P < 0.05, **P *<* 0.01, ***P < 0.001 versus model).

Because we demonstrated that blocking miR-155-5p ameliorated allergic inflammation in the AD model, we analyzed the effects of antagomir-155-5p on PKIα and TJ proteins in the elicitation stage (Fig. 6a) and initial stage of the AD model (Fig. 6b). The level of miR-155-5p was upregulated in the model and significantly reduced in the antogomir-155-5p-treated group in the elicitation stage, as previously shown (Fig. 2d), and in the initial stage of the AD model (Fig. 6h). Immunohistochemical staining indicated relatively lower expression of PKIα, CLDN-1, and occludin in biopsy specimens from the model mice, and antagomir-155-5p clearly increased the expression of PKIα, CLDN-1, and occludin in the elicitation stage (Fig. 6c). Clearly increased protein levels of PKIα, CLDN-1, and occludin proteins were detected in the antogomir-155-5p-treated group (Fig. 6d–g). We also found similar changes in the initial stage of AD (Fig. 6i–l). These results indicated that the expression of PKIα and the epithelial TJ proteins was regulated by miR-155-5p, not only in the elicitation stage but also in the initial stage of the AD model.

The epithelial-derived cytokines TSLP and IL-33 are key factors in the initiation of allergic inflammation. Because the miR-155-5p inhibitor was shown to play a role in the sensitization period, we next investigated its effects on TSLP and IL-33 expression in the initial stage of the AD model. Enhanced expression of TSLP and IL-33 was detected in the ear homogenates of the model mice, whereas its marked downregulation was observed in the antogomir-155-5p-treated group (Fig. 6m, n).

### Epithelial TJ proteins, TSLP, and IL-33 are regulated by PKIα

**Fig. 7:**
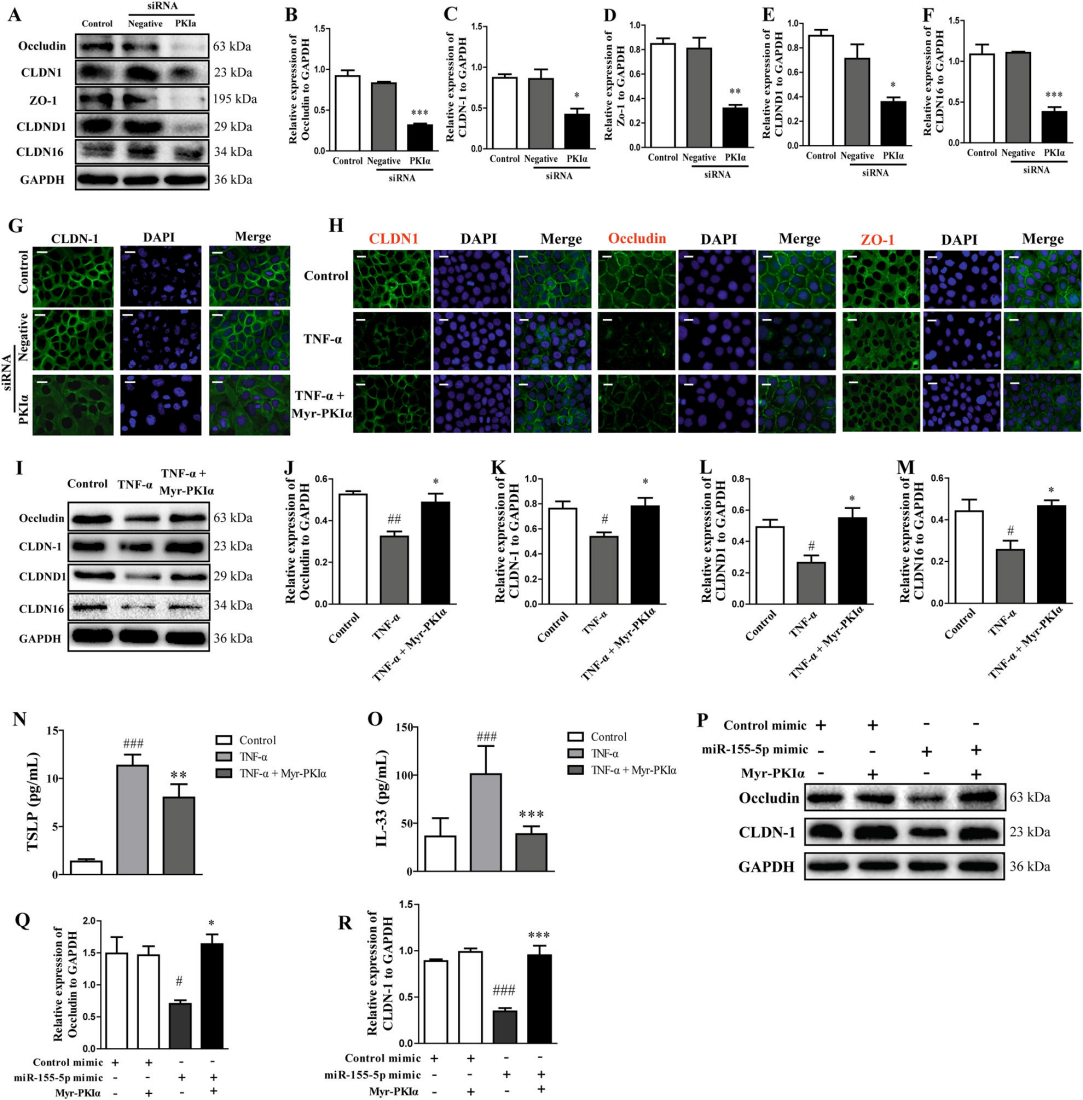
PKIα increased the expression of TJ proteins and reduced TSLP and IL-33 production in HaCaT cells. **a**–**f** PKIα, CLDN-1, occludin, ZO-1, CLDND1, and CLDN16 expression was analyzed with western blotting after cells were treated with PKIα siRNA. The expression was quantified relative to GAPDH expression with a ChemiScope analysis (means ± SD; *n* = 3; *P < 0.05, **P < 0.01, ***P < 0.001 versus negative siRNA). **g** Immunofluorescent quantification of CLDN-1 expression (*n* *=* 3; magnification: ×200; scale bar: 100 μm). **h** Immunofluorescent quantification of CLDN-1, occludin, and ZO-1 expression (*n* *=* 3; magnification: ×200; scale bar: 100 μm). **i–m** CLDN-1, occludin, CLDND1, and CLDN16 expression was analyzed with western blotting and quantified relative to GAPDH expression with a ChemiScope analysis (*n* = 3). **n**, **o** TSLP and IL-33 production was measured with ELISAs (means ± SD; *n* *=* 6; ^###^*P* < 0.001 versus control, **P *<* 0.01, ***P < 0.001 versus TNF-α). **p–r** CLDN-1 and occludin expression was analyzed with western blotting and quantified relative to GAPDH expression with a ChemiScope analysis (means ± SD; *n* *=* 3; ^#^*P* < 0.05, ^###^*P* < 0.001 versus control, *P < 0.05, ***P < 0.001 versus miR-155-5p mimic).

To investigate the involvement of PKIα in the regulation of TJs, HaCaT cells were treated with siRNA directed against PKIα. When the cells were transfected with the PKIα siRNA, PKIα expression was greatly reduced. The inhibition of PKIα resulted in significant reductions in CLDN-1, occludin, ZO-1, and CLDND1 (Fig. 7a–g). To clarify the biological role of the interaction between PKIα and TJs, TSLP, and IL-33, HaCaT cells were treated with myristoylated PKIα (Myr-PKIα; an analog of endogenous PKIα) for 6 h, and simultaneously with 20 ng/mL TNF-α for 24 h. Immunofluorescent staining showed that the cells treated with Myr-PKIα tended to show higher expression and normal distributions of CLDN-1 and occludin compared with those in the TNF-α-stimulated cells (Fig. 7h). Marked reductions in CLDN-1, occludin, CLDN16, and CLDND1 expression were detected with western blotting in the HaCaT cells treated with TNF-α, whereas Myr-PKIα significantly increased the expression of CLDN-1, occludin, CLDND1, and CLDN16 (Fig. 7i–m). The levels of TSLP and IL-33 proteins were effectively downregulated by Myr-PKIα (Fig. 7n, o). HaCaT cells were transfected with the miR-155-5p mimic and pretreated with Myr-PKIα to investigate whether PKIα can overcome the effects of increased microRNA-155-5p expression. The expression levels of CLDN-1 and occludin were detected with western blotting. The results showed that the overexpression of miR-155-5p markedly reduced the expression of CLDN-1 and occludin, which was restored by Myr-PKIα (Fig. 7p–r).

## Discussion

AD has become a major health problem because its prevalence is increasing, it affects the quality of life, and it plays a role in the progression to other atopic diseases^1,2^. Because AD is a chronic disease with no cure, effective preventive measures are sought^31,32^.

MiRNAs constitute an increasingly recognized group of regulatory molecules with important roles in cell fates^11^. Therefore, miRNAs are of considerable interest in understanding the immune regulation of allergic diseases^15^. In the present study, we demonstrated that miR-155-5p was upregulated in a model of AD. Treatment of the AD model with an miR-155-5p inhibitor before FITC sensitization significantly reduced the expression of miR-155-5p. Silencing miR-155-5p attenuated the thickening of the epidermis in AD and reduced the infiltration of inflammatory cells and the secretion of Th2 cytokines. These findings indicated that miR-155-5p was a critical regulator of AD-associated inflammation.

In terms of allergic disease, recent studies have demonstrated that miR-155-5p is overexpressed in AD and may be involved in its pathogenesis by modulating the differentiation and function of Th17 cells^33^. miR-155-5p is also essential for allergen-induced eosinophilic inflammation in the lung^18^. miR-155-5p may also exert a feedback effect upon the M1/M2 balance in macrophages^34^. Johansson et al. have recently demonstrated that miR-155-5p is expressed in type 2 innate lymphoid cells (ILC2s) and is a critical regulator of allergic airway inflammation^21^. miR-155-5p is also expressed in Th2 cells and is involved in the regulation of the Th2 responses in eosinophilic airway inflammation^18^. Therefore, the reduction in type 2 cytokine expression observed in our study when miR-155-5p was blocked by antagomir treatment might be dependent on several different types of cells involved in the inflammatory response. However, in the present study, we predominantly detected miR-155-5p-expressing cells in the mouse epidermis with tissue fluorescence in situ hybridization. To further clarify this, we separated the epidermis from the mouse ear, and showed that the expression of miR-155-5p was significantly increased in the epidermis. Plank et al. have demonstrated that an administered antagomir is quickly internalized by cells, but the uptake efficiency varies greatly across different cell types. Myeloid cells (including macrophages and neutrophils) show higher levels of antagomir uptake than lymphocytes. Notably, these authors found that lung epithelial cells also displayed good uptake of antagomir-155-5p^20^. Their and our studies indicate that epithelial cells take up the miR-155-5p antagomir, and may therefore play a functional role in AD. Consequently, we focused on the role played by miR-155-5p in epithelial cell functions.

The miRanda and TargetScan databases were used to predict the potential targets of miR-155-5p. We found that some targets of miRNA-155-5p were downregulated in the elicitation phase of the AD recurrence model. These predicted targets included PKIα, fibroblast growth factor 7 (FGF7), basonuclin 2 (BNC2), brain-specific angiogenesis inhibitor 1 (BAI1)-associated protein 2-like 1 (BAIAP2l1), HMG-box transcription factor 1 (HBP1), and olfactomedin-like protein 3 (OLFML3)^27^. PKIα was the target with the highest scores in the databases. The highest scores for target prediction in the databases screened indicated that PKIα mRNA interacts with miR-155-5p. Moreover, a previous study showed that protein kinase inhibitors prevent the disruption of epithelial TJs^30^. A luciferase reporter assay confirmed that PKIα mRNA was a specific target of miR-155-5p. PKIα is expressed in the human heart, brain, skin, and 11 other tissues^28^, but the role of PKIα in AD was unclear. The expression of PKIα correlated negatively with that of miR-155-5p in the AD model. An immunohistochemical analysis suggested that PKIα was mainly expressed in epithelial cells. We demonstrated that miR-155-5p upregulation correlated with PKIα downregulation in HaCaT cells.

The loss of barrier function in AD enhances the penetration of allergens, microbes, and irritants^35^, thus leading to the release of TSLP and IL-33^36^. The TSLP and IL-33 derived from epithelial cells are master switches of allergic inflammation. Johansson et al. showed that miR-155-5p plays a regulatory role in the ILC2 subset, which affects the expression of the IL-33 receptor, IL-33 responsiveness, and IL-13 production, as well as the proliferative capacity of ILC2s, possibly because of defects in the GATA-3 function in experimental models of allergic airway inflammation^21^. Overexpressed miRNA-155-5p dysregulates intestinal epithelial ZO-1 and E-cadherin by dysregulating RhoA in severe acute pancreatitis^26^. miR-155-5p is also essential for the development of an eosinophil response, apparently via its capacity to inhibit the expression of the transcription factor PU.1, which is a negative regulator of Th2 cytokine production in the lung^18^. However, there are limited data on the functional role of miR-155-5p in the skin in AD, especially in the epithelium.

In this study, when cells were transfected with an miR-155-5p inhibitor, the expression of PKIα clearly increased, and the expression of occludin and CLDN16 was also restored, whereas TSLP was significantly reduced compared with that in the cells treated with the inhibitor control. We also observed significantly reduced expression of PKIα after miR-155-5p was overexpressed with an miR-155-5p mimic. The expression of CLDN-1, occludin, and CLDND1 was also reduced and TSLP and IL-33 expression was upregulated in the miR-155-5p-expressing cells. These results suggested that miR-155-5p regulated TJs and key factors by directly targeting PKIα expression. Consistent with this, an immunohistochemical analysis showed the increased expression of epithelial PKIα and TJ proteins when miR-155-5p was inhibited in the AD mouse model and in epithelial cells. In the initial stage of AD, a lack of miR-155-5p increased the levels of PKIα and TJ proteins, but markedly inhibited the expression of TSLP and IL-33.

Recent studies have implied the involvement of protein kinase inhibitors in TJs. The disruption of epithelial occludin and ZO-1 was prevented by cyclic-nucleotide-dependent protein kinase inhibitors^30^. PKIα siRNA and Myr-PKIα were used to confirm that PKIα regulates TJs in HaCaT cells. This experiment showed that the expression of TJ proteins decreased when the cells were transfected with PKIα siRNA. Myr-PKIα clearly increased the expression of TJ proteins and significantly reduced the levels of TSLP and IL-33.

PKIα may exert its effect on TJs via several pathways, including by targeting PKA and regulating the importation of transcription factors. Because PKIα is a highly specific inhibitor of PKA, it may modulate cAMP-dependent PKA signaling^37,38^. It has been suggested that the activation of PKA plays a role in TJ disruption. The TJ localization of CLDN16 is regulated by cAMP/PKA-dependent phosphorylation^39^. The phosphorylation of CLDN-3 at threonine 192 by a cAMP-dependent PKA regulates the TJ barrier function^40^. In addition to the negative regulation of cyclin- or cAMP-dependent protein kinase activity, PKIα could negatively regulate protein importation into the nucleus, further influencing downstream molecules^41^. Therefore, PKIα may exert its influence on TJ proteins by regulating the entry of key transcription factors into the nucleus. Future studies will be required to better understand how PKIα regulates TJ proteins.

**Fig. 8:**
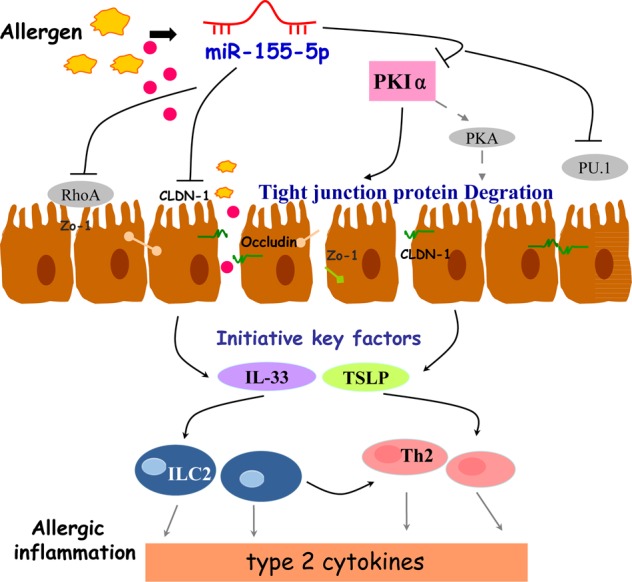
Schematic for the contribution of miR-155-5p to the pathogenesis of allergy. Other studies have reported that miR-155-5p dysregulates intestinal epithelial Zo-1 by targeting RhoA. In addition, miR-155-5p regulates Th2 cytokines by inhibiting the expression of the PU.1 in the lung. In this study, upregulation of miR-155-5p directly targeting PKIα, which induces the disruption of TJ proteins, and then increases the expression of initiative key factors (TSLP, IL-33), promoted the release of type 2 cytokines and aggravated the allergic inflammation.

In summary, we have demonstrated for the first time that miR-155-5p was critical for the allergic inflammation in a mouse model of AD. We have clearly shown that miR-155-5p had a previously unknown direct role in regulating PKIα expression, which affected the epithelial expression of TJ proteins (Fig. 8). These findings may lead to new therapeutic strategies and targets for the treatment of allergic disorders with a disturbed skin barrier, by the regulation of miR-155-5p. Our data also opened new avenues that should extend our understanding of the basic miRNA mechanisms involved in the onset and maintenance of allergic inflammation in AD.

## Materials and Methods

### Animals and cells

Male BALB/c mice were purchased from Shanghai SLAC Laboratory Animal (Shanghai, China). All animals were maintained at Nanjing University of Chinese Medicine under specific pathogen-free conditions at 18–25 °C and 50–60% humidity, and were used at 6–8 weeks of age. The animals were allocated to experimental groups with weight-stratified randomization. The investigator was blinded to the group allocation during the experiment. All procedures involving animals were approved by the Animal Care and Use Committee of Nanjing University of Chinese Medicine (ACU-16, 24-11-2015) and were performed strictly according to the Guide for the Care and Use of Laboratory Animals. HaCaT cells (immortalized human epidermal cells) were purchased from the Cell Bank of the Chinese Academy of Medical Sciences (Beijing, China). The cells were identified with short tandem repeat profiling and tested for mycoplasma contamination.

### Mouse model of FITC-induced AD and miR-155-5p inhibitor treatment in vivo

To induce AD-like inflammation, 1.5% FITC in 80 μL of a mixture of acetone and dibutylphthalate (1:1, vehicle) was applied to the abdominal skin of the BALB/c mice on days 1 and 2, and their right ears were treated with 0.6% FITC solution on day 6. Acetone and dibutylphthalate mixture was used as the vehicle control (control group; *n* = 7)^9^. The mice in the antagomir-155-5p group (*n* = 7) were subcutaneously injected once daily with an miR-155-5p inhibitor (5 nmol/10 μL; micrOFF mmu-miR-155-5p antagomir; RiboBio, Guangzhou, China). The mice in the control group and model group (*n* = 7 each) were subcutaneously injected with the scrambled antagomir negative control (5 nmol/10 μL; micrOFF antagomir negative control; RiboBio) for three consecutive days, on days −1 to 1. Ear thickness measurements were made with a thickness gauge (Mitutoyo, Kawasaki, Japan) on day 7, after elicitation for 24 h. The murine ear skin tissues were fixed in 4% paraformaldehyde (PFA) and embedded in paraffin before sectioning and staining with hematoxylin and eosin (H&E). The numbers of inflammatory cells were quantified with the Mantra Quantitative Pathology Workstation (PerkinElmer, Waltham, MA, USA).

### Cell culture in vitro

HaCaT cells were cultured in minimal essential medium (Thermo Fisher Scientific, Waltham, MA, USA) supplemented with 10% fetal bovine serum (Capricorn Scientific, Hessen, Germany) at 37 °C in 5% CO_2_. The cells were treated with or without TNF-α (20 ng/mL; R&D Systems, Minneapolis, MN, USA) for 24 h. HaCaT cells were pretreated with or without myristoylated PKIα (Myr-PKIα, 2 μM; Merck Millipore, Billerica, MA, USA) for 6 h and simultaneously stimulated with TNF-α for 24 h.

### The miRNA target prediction and verification with luciferase reporter

The miRanda and TargetScan databases were used to predict the potential targets of miR-155-5p. A luciferase reporter assay was performed with the Dual-Luciferase Reporter Assay System with the wild-type PKIα-3′UTR and mut-PKIα-3′UTR dual luciferase reporter vectors (Promega, Madison, WI, USA) synthesized and tested by Hanbio Biotechno (Shanghai, China). 293T cells were then cotransfected with one or the other of these plasmids and an miR-155-5p mimic. Cells were also transfected with the control vector to monitor the transfection efficiency. We also included an miR-155-5p negative control and an miRNA with no homolog in the human genome as a control. After 48 h, the firefly luciferase activity was determined with the Dual-Luciferase Reporter Assay System (Promega) in a microplate system (Tecan, Männedorf, Switzerland). We calculated the relative reporter activity by normalization to the *Renilla* control.

### Fluorescence in situ hybridization

Paraffin-embedded 4%-PFA-fixed ear tissues were cut into 6 µm sections and deparaffinized. The antigen was retrieved by boiling in citric acid buffer in a water bath for 20 min. Proteinase K (200 μL; Servicebio, Wuhan, China) in PBS was added to the sections in a humidified chamber, which were then incubated for 25 min at 37 °C and washed twice with PBS for 5 min each. Prehybridization buffer (100 µL; Servicebio) was added to each tissue section. The sections were placed in a hybridization chamber, incubated for 1 h at 37 °C. The prehybridization buffer was replaced with hybridization buffer containing the FAM-labeled miR-155-5p probe (5′-ACCCCTATCACAATTAGCATTAA-3′; Servicebio). The tissues of the negative control mice were incubated in hybridization buffer without the probe to exclude nonspecific staining; the other steps were the same as in the control and model groups. The samples were allowed to hybridize overnight at 37 °C. DAPI (Servicebio) was used for nuclear staining.

### Epidermal separation

The murine ear skin tissues were divided into two pieces and incubated dermis-side-down in 0.125% dispase in PBS for 2 h at 37 °C. The tissues were washed with PBS and the epidermis was carefully peeled off the dermis.

### Transfection with miR-155-5p inhibitor or mimic

HaCaT cells were seeded in 6-well or 12-well plates at a density of 1 × 10^5^ cells/mL. At 50% confluence, the cells were transfected with 50 nM micrOFF miR-155-5p (5′-ACCCCUAUCACAAUUAGCAUUAA-3′) or the inhibitor control, or with micrON miR-155-5p (miR-155-5p mimic; sense: 5′-UUAAUGCUAAUUGUGAUAGGGGU-3′; antisense: ACCCCUAUCACAAUUAGCAUUAA) or the mimic control (RiboBio) using Lipofectamine 2000 (Life Technologies Corporation, Gaithersburg, MD, USA), according to the manufacturer’s instructions. The RNA–lipid complexes were added to the HaCaT cells, and the medium was replaced after 6 h. After the cells were transfected for 48 h, they were stimulated with TNF-α for 12 h.

HaCaT cells were seeded in six-well plates at a density of 1 × 10^5^ cells/mL. At 50% confluence, the cells were transfected with 50 nM micrON miR-155-5p (miR-155-5p mimic) or the mimic control. The RNA–lipid complexes were added to the HaCaT cells, and the medium was replaced after 6 h. After the cells were transfected for 24 h, they were treated with or without Myr-PKIα (2 μM) for 24 h.

### Transfection with PKIα siRNA

HaCaT cells were seeded in 6-well or 12-well plates at a density of 1 × 10^5^ cells/mL. The cells were transfected with 50 nM PKIα or negative siRNA (Transheep) using Lipofectamine 2000, according to the manufacturer’s instructions. The siRNA–lipid complexes were added to the HaCaT cells, and the medium was replaced after 6 h. After transfection for 48 h, the samples were collected for analysis.

### Measurement of cytokines

The concentrations of IL-4, IL-5, IL-9, and IL-13 in the ear homogenates and TSLP and IL-33 in cell culture supernatants were measured with enzyme-linked immunosorbent assay (ELISA) kits (eBioscience, San Diego, CA, USA), according to the manufacturer’s instructions. The total protein levels in the homogenates were measured with a Bicinchoninic acid (BCA) protein assay kit (Thermo Fisher Scientific). The cytokine protein levels were calculated with the formula: concentration of cytokine in the homogenate/total protein in the homogenate (pg/mg).

### Reverse transcription-quantitative real-time PCR

Total RNA was isolated from the ear tissues or cells with TRIzol Reagent (Life Technologies Corporation). cDNA was synthesized with an oligo(dT) primer and SuperScript II RT (Invitrogen, Carlsbad, CA, USA). Gene expression levels were determined with the ABI 7500 Fast Real-Time PCR System (Applied Biosystems, Carlsbad, CA, USA) using the SYBR Green PCR Master Mix (Thermo Fisher Scientific). The Bulge-loop miRNA qRT–PCR Primer Sets (one RT primer and a pair of qPCR primers in each set) specific for miR-155-5p and U6 were designed by RiboBio. The mRNA primer sequences (GenScript, Nanjing, China) used for RT–qPCR were mouse PKIα: 5′-AGAGAAGCTCCACCGAACAA-3′ (forward, F), 5′-TGGCAACCAACAGTGTCTTG-3′ (reverse, R); human PKIα: 5′-GTGTGGTTGTGCCAGAAACT-3′ (F), 5′-GCAACCATGCCCTTATTCCA-3′ (R); human IL-33: 5′-CGGTGTTGATGGTAAGATG-3′ (F), 5′-AGAGTGTTCCTTGTTGTTG-3′ (R); mouse glyceraldehyde 3-phosphate dehydrogenase (GAPDH): 5′-GGTTGTCTCCTGCGACTTCA-3′ (F), 5′-TGGTCCAGGGTTTCTTACTCC-3′ (R); human GAPDH: 5′-CTTCTTTTGCGTCGCCAGCCGA-3′ (F), 5′-ACCAGGCGCCCAATACGACCAA-3′ (R). Gene expression was normalized to GAPDH expression and relative expression was calculated with the ΔΔCt method.

### Western blotting analysis

Ear tissues of mice were ground into homogenates and the cells were scraped with protein lysis solution (RIPA:phenylmethylsulfonyl fluoride = 100:1). The samples were collected in microcentrifuge tubes and lysed for 30 min. The protein concentrations of the samples were determined with a BCA protein assay kit (Thermo Fisher Scientific). Total protein extracts were resolved with SDS–PAGE and transferred onto polyvinylidene difluoride membranes (Merck Millipore). The membranes were blocked with 5% nonfat dry milk in Tris-buffered saline containing 0.1% Tween 20 (TBST) for 1 h and washed five times for 5 min each with TBST at room temperature. The samples were then incubated overnight at 4 °C with antibodies directed against PKIα (1:1,000 dilution; Santa Cruz Biotechnology, Santa Cruz, CA, USA; sc-50349), claudin 1 (CLDN-1; ab15098), occludin (ab168986), claudin domain containing 1 (CLDND1; ab80440), or claudin 16 (CLDN16; ab106504) (1:1,000 dilution; Abcam, Cambridge, MA, USA). After the membranes were washed, they were incubated with a secondary peroxidase-linked goat anti-rabbit IgG antibody (1:1,000 dilution; Santa Cruz Biotechnology) for 2 h at room temperature. The protein bands were detected with Immobilon Western Chemiluminescent HRP Substrate (Merck Millipore) and protein expression was quantified with gel analysis software.

### Immunohistochemistry

Tissue sections (6 μm) were fixed successively in dimethylbenzene, 100% ethanol, 95% ethanol, 80% ethanol, and 75% ethanol. The antigen was retrieved by heating the samples in citric acid buffer at 95 °C in a water bath for 20 min, and then cooling them to room temperature. Endogenous peroxidase was blocked with 3% H_2_O_2_ and nonspecific binding sites were blocked with 5% bovine serum albumin (BSA). The sections were immunostained with a rabbit monoclonal antibody directed against PKIa (1:100 dilution; Santa Cruz Biotechnology), CLDN-1, or occludin (1:100 dilution; Abcam) at 4 °C overnight. After repeated washes with PBS, the sections were incubated with a biotinylated secondary antibody (Zhongshanjinqiao, Beijing, China), then developed in prepared 3,3′-diaminobenzidine (DAB) chromogenic solution. The samples were lightly counterstained with hematoxylin. The slides were dehydrated in a graded series of ethanol (75%, 80%, 95%, and 100%), and finally washed with dimethylbenzene. The specimens were mounted and analyzed with optical microscopy (Axion A1, Carl Zeiss AG, Germany). The mean DAB intensity was quantified with a Mantra Quantitative Pathology Workstation (PerkinElmer).

### Immunofluorescence

The cells on the coverslips were washed with PBS before they were fixed with methanol for 20 min at −20 °C. Nonspecific binding sites were blocked with 1.5% BSA for 1 h at room temperature. The cells were incubated with a rabbit antibody directed against PKIa (1:100 dilution), CLDN-1, occludin, or ZO-1 (1:100 dilution; Abcam; ab96587) at 4 °C overnight. After the cells were washed with PBS, they were stained with a FITC-conjugated goat anti-rabbit IgG antibody (1:200; dilution; Santa Cruz Biotechnology) and DAPI (Bioword, Nanjing, China) at a concentration of 0.1 μg/mL for 10 min in the dark. The labeled sections were observed with fluorescence optical microscopy (PerkinElmer).

### Statistical analysis

The data are expressed as means ± standard deviations (SD). Multiple groups were compared with one-way analysis of variance, and Dunnett’s test was used to compare two groups, with GraphPad Prism 5 (GraphPad Software, USA). All experiments were repeated three times. Statistical significance was set at *P* *<* 0.05.

## References

[1] Wollenberg A, Feichtner K (2013). Atopic dermatitis and skin allergies - update and outlook. Allergy.

[2] Kramer ON, Strom MA, Ladizinski B, Lio PA (2017). The history of atopic dermatitis. Clin. Dermatol..

[3] Leung DY (2016). Clinical implications of new mechanistic insights into atopic dermatitis. Curr. Opin. Pediatr..

[4] Holgate ST (2007). Epithelium dysfunction in asthma. J. Allergy Clin. Immunol..

[5] Xiao C (2011). Defective epithelial barrier function in asthma. J. Allergy Clin. Immunol..

[6] Salim SY, Soderholm JD (2011). Importance of disrupted intestinal barrier in inflammatory bowel diseases. Inflamm. Bowel Dis..

[7] De Benedetto A (2011). Tight junction defects in patients with atopic dermatitis. J. Allergy Clin. Immunol..

[8] Holgate ST (2007). The epithelium takes centre stage in asthma and atopic dermatitis. Trends Immunol..

[9] Wang X (2017). Cimifugin suppresses allergic inflammation by reducing epithelial derived initiative key factors via regulating tight junctions. J. Cell. Mol. Med..

[10] Jungersted JM (2010). Stratum corneum lipids, skin barrier function and filaggrin mutations in patients with atopic eczema. Allergy.

[11] Wilczynska A, Bushell M (2015). The complexity of miRNA-mediated repression. Cell Death Differ..

[12] Mehta A, Baltimore D (2016). MicroRNAs as regulatory elements in immune system logic. Nat. Rev. Immunol..

[13] Thai TH (2007). Regulation of the germinal center response by microRNA-155. Science.

[14] Xiao C, Rajewsky K (2009). MicroRNA control in the immune system: basic principles. Cell.

[15] Rebane A, Akdis CA (2013). MicroRNAs: essential players in the regulation of inflammation. J. Allergy Clin. Immunol..

[16] Lu TX, Rothenberg ME (2013). Diagnostic, functional, and therapeutic roles of microRNA in allergic diseases. J. Allergy Clin. Immunol..

[17] Sonkoly E, Stahle M, Pivarcsi A (2008). MicroRNAs: novel regulators in skin inflammation. Clin. Exp. Dermatol..

[18] Malmhall C (2014). MicroRNA-155 is essential for T(H)2-mediated allergen-induced eosinophilic inflammation in the lung. J. Allergy Clin. Immunol..

[19] Zech A (2015). MicroRNA-155 modulates P2R signaling and Th2 priming of dendritic cells during allergic airway inflammation in mice. Allergy.

[20] Plank MW (2015). MicroRNA expression is altered in an ovalbumin-induced asthma model and targeting miR-155 with antagomirs reveals cellular specificity. PLoS ONE.

[21] Johansson K, Malmhall C, Ramos-Ramirez P, Radinger M (2017). MicroRNA-155 is a critical regulator of type 2 innate lymphoid cells and IL-33 signaling in experimental models of allergic airway inflammation. J. Allergy Clin. Immunol..

[22] Okoye IS (2014). Transcriptomics identified a critical role for Th2 cell-intrinsic miR-155 in mediating allergy and antihelminth immunity. Proc. Natl Acad. Sci. USA.

[23] Sonkoly E (2010). MiR-155 is overexpressed in patients with atopic dermatitis and modulates T-cell proliferative responses by targeting cytotoxic T lymphocyte-associated antigen 4. J. Allergy Clin. Immunol..

[24] Suojalehto H (2013). MicroRNA profiles in nasal mucosa of patients with allergic and nonallergic rhinitis and asthma. Int. Forum Allergy Rhinol..

[25] Qin W, Ren Q, Liu T, Huang Y, Wang J (2013). MicroRNA-155 is a novel suppressor of ovarian cancer-initiating cells that targets CLDN1. FEBS Lett..

[26] Tian R, Wang RL, Xie H, Jin W, Yu KL (2013). Overexpressed miRNA-155 dysregulates intestinal epithelial apical junctional complex in severe acute pancreatitis. World J. Gastroenterol..

[27] Wang X (2018). Integrative analysis of lncRNAs, miRNAs, and mRNA-associated ceRNA network in an atopic dermatitis recurrence model. Int. J. Mol. Sci..

[28] Fagerberg L (2014). Analysis of the human tissue-specific expression by genome-wide integration of transcriptomics and antibody-based proteomics. Mol. Cell. Proteomics.

[29] Esaki H (2015). Identification of novel immune and barrier genes in atopic dermatitis by means of laser capture microdissection. J. Allergy Clin. Immunol..

[30] Klingler C (2000). Disruption of epithelial tight junctions is prevented by cyclic nucleotide-dependent protein kinase inhibitors. Histochem. Cell Biol..

[31] Weidinger S, Novak N (2014). Atopic dermatitis revisited. Allergy.

[32] Dharmage SC (2014). Atopic dermatitis and the atopic march revisited. Allergy.

[33] Ma L, Xue HB, Wang F, Shu CM, Zhang JH (2015). MicroRNA-155 may be involved in the pathogenesis of atopic dermatitis by modulating the differentiation and function of T helper type 17 (Th17) cells. Clin. Exp. Immunol..

[34] Martinez-Nunez RT, Louafi F, Sanchez-Elsner T (2011). The interleukin 13 (IL-13) pathway in human macrophages is modulated by microRNA-155 via direct targeting of interleukin 13 receptor alpha1 (IL13Ralpha1). J. Biol. Chem..

[35] Sugawara T (2013). Tight junction dysfunction in the stratum granulosum leads to aberrant stratum corneum barrier function in claudin-1-deficient mice. J. Dermatol. Sci..

[36] Hammad H, Lambrecht BN (2015). Barrier epithelial cells and the control of type 2 immunity. Immunity.

[37] Kazgan N, Williams T, Forsberg LJ, Brenman JE (2010). Identification of a nuclear export signal in the catalytic subunit of AMP-activated protein kinase. Mol. Biol. Cell.

[38] Kawakami M, Nakanishi N (2001). The role of an endogenous PKA inhibitor, PKIalpha, in organizing left-right axis formation. Development.

[39] Ikari A (2008). Claudin-16 is directly phosphorylated by protein kinase A independently of a vasodilator-stimulated phosphoprotein-mediated pathway. J. Cell. Physiol..

[40] D’Souza T, Agarwal R, Morin PJ (2005). Phosphorylation of claudin-3 at threonine 192 by cAMP-dependent protein kinase regulates tight junction barrier function in ovarian cancer cells. J. Biol. Chem..

[41] Dalton GD, Dewey WL (2006). Protein kinase inhibitor peptide (PKI): a family of endogenous neuropeptides that modulate neuronal cAMP-dependent protein kinase function. Neuropeptides.

